# Comparative Efficacy of Topical Pertmehrin, Crotamiton and Sulfur Ointment in Treatment of Scabies

**Published:** 2017-03-14

**Authors:** Celestyna Mila-Kierzenkowska, Alina Woźniak, Ewa Krzyżyńska-Malinowska, Lucyna Kałużna, Roland Wesołowski, Wojciech Poćwiardowski, Marcin Owcarz

**Affiliations:** 1 The Chair of Medical Biology, Collegium Medicum of Nicolaus Copernicus University, Bydgoszcz, Poland; 2 Department of Cosmetology, Torun Higher School of Business, Toruń, Poland; 3 Department of Cosmetology and Aesthetic Dermatology, Collegium Medicum of Nicolaus Copernicus University, Bydgoszcz, Poland; 4 Institute of Food Technology, Faculty of Chemical Technology and Engineering, University of Technology and Life Sciences, Bydgoszcz, Poland; 5 Ludwik Rydygier Voivodship Polyclinical Hospital, Toruń, Poland

**Keywords:** Acaricides, Skin diseases, Parasitic infection

## Abstract

**Background:**

Scabies is an ectoparasitic infection, which occurs because of direct skin-to skin contact. The ideal treatment modality is still unclear and further research on this topic is warranted. The aim of the study was to compare the efficacy and safety of the topical scabicides: permethrin, crotamiton and sulfur ointment.

**Methods:**

Fifty four patients with diagnosed scabies were randomly divided into three treatment groups. The first group received 5% permethrin cream twice with one week interval, the patients from the second group were given crotamiton lotion for two days twice with one week interval, while the third group received 10% sulfur ointment for two or three weeks. All patients were followed up at 1, 2 and 4 weeks intervals.

**Results:**

At one-week follow up the cure rate was significantly higher at permethrin-treated group when compared to crotamiton group (P< 0.001) and sulfur group (P< 0.001). At the end of two-week interval, the cure rate at permethrin group was 100%, while at crotamiton group, 66.7% and in sulfur group 38.9% (P< 0.001). At 4-week follow up the applied treatment was effective in all studied individuals.

**Conclusion:**

The topical application of permethrin, crotamiton and sulfur was equally efficacious at 4-week follow up, however permethrin cream showed faster improvement at first and second follow up. Acquiring permethrin is considered as expensive option and crotamiton lotion seems to be cost-less alternative to this cream.

## Introduction

Scabies is a contagious parasitic dermatitis that occurs among humans and other animals. The disease is caused by a tiny and usually not directly visible parasite, the mite *Sarcoptes scabiei* (Goldust et al. 2013a). Scabies appears worldwide and is considered significant public health problem in the developing world. There are over 300 million cases of scabies reported annually worldwide (Arif Maan et al. 2015, Thomas et al. 2015). The prevalence of scabies ranges from 2.2% in European and Middle Eastern countries to 71% in Papua New Guinea and the highest incidence of this infection is observed in the Pacific and Latin American regions as well as in aboriginal communities in northern Australia (McLean 2013, Romani et al. 2015). Scabies affects regardless age, gender, race and social class, however, risk factors include poverty, poor nutritional status, homeless, dementia and poor hygiene (Shimose and Munoz-Prize 2013). The transmission occurs via direct skin-to-skin contact with an infected individual and it usually takes 15 to 20 min of close contact for successful transfer the mite to another person (Chosidow 2012).

The symptoms of scabies usually emerge up to 4 weeks following initial infestation (Shimose and Munoz-Prize 2013). Human scabies is characterized clinically by pruritus with nocturnal exacerbation and scabietic nodules, and visible skin burrows can be the pathognomic lesions of scabies (Hicks and Elston 2009). Classic locations of burrows are the interdigital spaces of the hand, the flexural surface of the wrist, elbows, genitalia, axillae, umbilicus, belt line, nipples, buttock, and penis shaft. Among pediatric population, scabies can also affect the head, neck, face, palm and soles (Andrews et al. 2009).

There are few methods to diagnose the scabies to be sufficiently sensitive, cost effective and convenient. The “gold standard” for diagnosis of human scabies with 100% specificity is the identification of mites, eggs, or feces from scrapings of infested skin or by identification of mite burrows (Walter et al. 2011). This method relies on physically locating parasite on the host, so it can have low sensitivity when mites are low in number. Alternate diagnosis method for scabies include polymerase chain reaction (PCR), microscopic examination of KOH prepared skin scrapings and dermoscopy, but they are still not easily applied to clinical or public health settings (Fukuyama et al. 2010, Park et al. 2012, Golant and Levitt 2012). No commercial immunodiagnostic tests for human scabies are currently available, and existing animal tests are not sufficiently sensitive (Rampton et al. 2013).

Treatment of scabies is a relevant issue in infectious dermatology and is as important as making correct diagnosis. Scabies is commonly treated with various medications called acaricides, but the treatment of choice is still controversial and the search for ideal scabicide is ongoing. An ideal medication should be effective against adults and eggs, easily applicable, non-irritating, non-toxic and economical. The mainstay of treatment of scabies is topical application of scabicidal agents, like permethrin 5% cream, lindane 1% lotion or cream, benzyl benzoate 10% and 20% lotion or emulsion, crotamiton 10% cream, precipitated sulphur 2–10% ointment, ivermectin 0,8 % cream and others (Karthikeyan 2005). Nowadays, oral administration of ivermectin is being increasingly used (Mounsey and McCarthy 2013). Despite the availability of effective therapeutics, treatment failures still occur, mostly secondary to application error or failure to decontaminate fomites (Golant and Levitt 2012). The aim of the study was to compare the efficacy and safety of topical 5% permethrin cream vs. crotamiton lotion and 10% sulfur ointment in the treatment of scabies.

## Materials and Methods

**Fig. 1.**
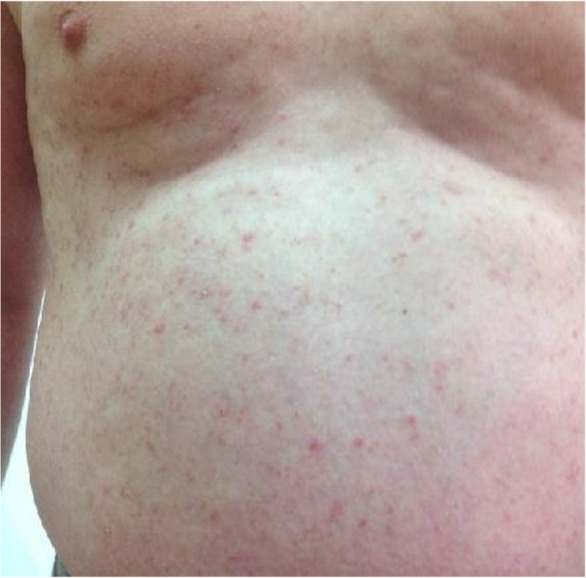
Characteristic skin lesions in adult with classic scabies

This single blind, randomized trial was conducted in patients with newly diagnosed scabies, of either gender, who were older than 18 years of age and voluntary agreed to participate in the study. Diagnosis of the disease was based on clinical symptoms and clinical history. For inclusion, the patients had to satisfy the four criteria, like the demonstration of classical burrows, presence of typical scabietic lesions at the classical sites (Fig. 1), complaint of nocturnal pruritus and family history of similar illness. Exclusion criteria included age under 18 years, history of allergy to any of the studied drugs, pregnancy or lactation, women planning for conception in near future as well as history of severe systemic disorders, like cardiac disorders, nervous system disorders, psychiatric illness and immunosuppressive disorders. Participants with abnormal kidney and liver function and known chronic infectious diseases were also excluded.

Before entry to the study, patients were given a physical examination and their history of infestations; antibiotic treatment and other information were recorded. Participants with any other coexisting skin disease, which could interfere with subsequent monitoring of scabies and patients with atypical presentations, as crusted scabies were excluded from the trial. None of the patients participating in the trial had been treated with pediculicides, scabicides or other topical agents in the month preceding the trial.

The studied group consisted of total 54 patients (22 males and 32 females) from 19 to 83 years of age. The patients were allocated to any of three treatment groups with simple randomization. All participants received detailed information about proper administration of the used drug. The patients included into the first group (8 men and 10 women) received single application of 5% permethrin cream at Day 1 and they were asked to put a thin layer of cream to all areas of the body including the face and the scalp. The cream was washed off after 8 hours and the single application was repeated 1 week later if live mites were seen during one-week follow up. The second group (7 men and 11 women) was given topical crotamiton lotion and they were told to apply the drug to the entire skin surface, rinse off after 24 hours and then reapply for an additional 24 hours. The procedure was repeated at all individuals twice, with one-week interval. When there was no cure in two weeks, 2^nd^ treatment was given with 10% sulfur ointment. The third group (7 men and 11 women) received 10% sulfur ointment for the period of two or three weeks (if there were no cure at 2-week follow up). Ten percent precipitate sulfur in petroleum base was used and as other topical drugs, it was thoroughly rubbed into the skin over the whole body covering neck to toe. The patients were asked to wash off the ointment after 24 hours and then reapply the drug every 24 hours for two (or three) weeks with a bath taken between each application. The treatment was given to both patients and their close family members, even without symptoms, at the same time. The participants of the study were also asked not to use any antipruritic drug or any other topical medications.

On entry of the study baseline, clinical parameters were comparable and the number of patients in each treatment group who were graded as having mild, moderate or severe infestation was not statistically different. The clinical evaluation after treatment was made by experienced investigator who was blinded to the treatments received. Patients in all the three groups were followed up at intervals of 1, 2 and 4 weeks to assess compliance and to examine clinically the patients to evaluate efficacy and safety. At each assessment, the investigator recorded the count of the lesions and grading of pruritus both subjectively and objectively by the patients as described on the first visit. Any adverse events were also recorded. The patient was considered as “cured” in case of the absence of new lesions, clinical improvement in the skin lesions and the improvement in the pruritus assessed by the visual analogue scale. “Re-infestation” was defined as a cure at 2 weeks but development of new lesions at 1-month follow-up. Treatment would be considered as failure if at the end of 4 weeks there was no improvement in the skin lesions and pruritus.

The study was approved by the appropriate Bioethics Committee (number KB 135/2014) and written informed consent was obtained from all the patients.

The percentage of improvement was compared between groups using the χ^2^ test followed by post-hoc Tukey’s test and P< 0.05 was considered significant. SPSS software (IBM SPSS Statistics 21) (Chicago, IL, USA) was used for all the analysis.

## Results

**Fig. 2.**
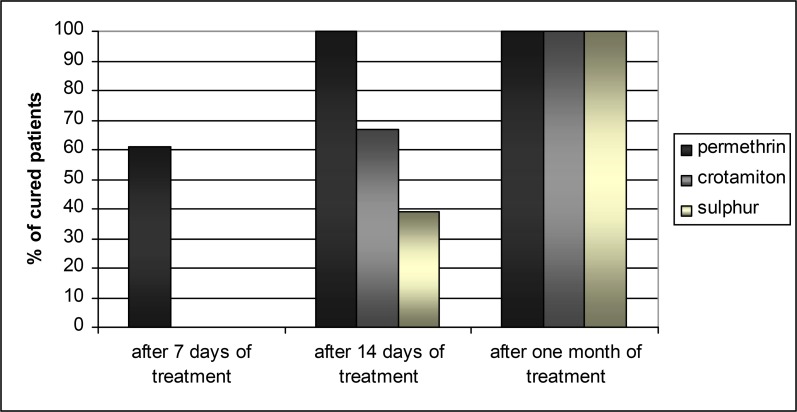
The cure rate at studied patients with scabies treated with 5% permethrin cream, crotamiton lotion and 10% sulphur ointment

At one-week follow up in permethrin treated group the treatment was effective in 11 of 18 patients (61.1%) and in none of patients in crotamiton and sulfur group (Fig. 2). Thus, at first follow up the cure rate was significantly higher after the single application of 5% permethrin cream when compared to the use of crotamiton lotion (P< 0.001) and 10% sulfur ointment (P< 0.001). At the end of two-week interval, the cure rate at permethrin group increased to 100% (after repeating application at remained 7 patients who had infestations at one-week follow up) and after two weeks of treatment none of patients included to this group still had severe itching and skin lesions. In crotamiton group, at the second follow-up, the cure rate was 66.7% (12 of 18 patients) compared with cure rate of 38.9% (7 of 18 patients) in the sulfur ointment group (P< 0.001). After two weeks of treatment the remaining 17 patients (6 in crotamiton group and 11 in sulfur group), who still manifested with scabietic lesions were treated with 10% sulfur ointment for the next week and the infestation was cured at all of them after three weeks of treatment (Fig. 2). Hence, at 4-week follow up the applied treatment was effective in all studied individuals.

All treatment modalities studied in this paper were considered cosmetically acceptable and well tolerated by all patients. None of participants experienced allergic reactions and no major adverse events were observed in any of the 3 groups. The main adverse event was skin dryness, reported by patients treated with sulphur ointment longer that two weeks, but it was not serious and did not affect compliance. None of the patients experienced worsening of infestation during the study, but at two patients treated with sulfur ointment re-infestation occurred at one-month follow-up.

## Discussion

Our study compared the use of 5% permethrin cream with that of crotamiton lotion and 10% sulfur ointment. The obtained results demonstrate that 5% permethrin cream is the most effective drug at 1 week follow up in treating scabies, what is in accordance with previous studies that have reported excellent cure rates with permethrin. In our study single application of permethrin resulted in improvement at 61.1% of patients, while in a study carried by Usha and Gopalakrishnan (2000) even a higher number of patients (97.8%) showed clearance of lesions at one-week follow up as compared to our results. Bachewar et al. (2009) found that at one week follow up, permethrin had significantly better cure rate than ivermectin, but at the end of two-weeks treatment this finding was reversed. Taplin et al. (1990) in a double-blinded, randomized study compared crotamiton 10% cream and permethrin 5% cream for the treatment of scabies in children 2 months to 5 years of age. Two weeks after a single overnight treatment, 30% children were cured with permethrin, in contrast to only 13% of subjects treated with crotamiton. Four weeks after treatment the efficacy in favor of permethrin was still statistically significant and this agent also demonstrated greater effectiveness in reducing pruritus and secondary bacterial infections. A single overnight topical application of 5% permethrin cream is superior to a single dose of oral ivermectin (Usha and Gopalakrishnan 2000). Permethrin being both miticidal and ovicidal appears to be more effective than for example crotamiton, which though is effective for adult stages of the mite, is not known to kill eggs and larvae. Thus, single application of crotamiton may be not accurate to eradicate the parasite and a second dose is needed within 1 to 2 weeks for 100% cure.

The study of Pourhasan et al. (2013) demonstrated, in turn, that in the treatment of scabies 5% permethrin cream was as effective as 10% crotamiton cream at two week follow-up. In our study at the second follow-up the cure rate for permethrin group was 100% and in crotamiton group 66.7%, while after 4 weeks, considered the definitive point for evaluating the efficacy of applied treatments, 5% permethrin cream was found to be as effective as crotamiton lotion and 10% sulfur ointment in treating scabies. This is in accordance with few reports about the comparable efficacy of treatment with permethrin cream and other scabicides, like topical 1% ivermectin (Goldust et al. 2013b), topical Tenutex emulsion (containing among others disulfiram and benzyl benzoate) (Goldust et al. 2013c) as well as oral ivermectin (Ranjkesh et al. 2013). Sharma and Singal (Sharma and Singal 2011) in randomized double-blind controlled study evaluated the efficacy and safety of topical 5% permethrin and oral ivermectin in single and two dose regimens in treatment of scabies. Their study revealed that all three-treatment modalities had the equal efficacy at the end of 4 weeks of treatment. Moreover, Chhaiya et al. (2012) reported that topical ivermectin as 1% lotion is as effective as 5% permethrin, but significantly more effective than oral ivermectin. On the other hand, Abedin et al. (2007) found that mass treatment of scabies with 2 doses of oral ivermectin in an endemic pediatric population is more efficacious than single topical application of 5% permethrin. Oral ivermectin produced also significantly better cure rate than lindane 1% lotion at 4 weeks follow-up (Mohhebipour et al. 2013). The authors suggest that oral ivermectin can be alternative treatment at patients with scabies, for whom topical therapies can cause serious cutaneous and systemic problems. Ivermectin is generally considered as effective as permethrin and more effective than other medications, such as lindane, benzyl benzoate, crotamiton and malathion (Panahi et. 2015). Moreover, Miyajima et al. (2015) described a novel method for scabies treatment called “whole-body bathing method”. In this method, the patients would bathe themselves in a fluid containing ivermectin at an effective concentration.

The selection of a drug is often based on the personal preference of physician, local availability and cost for the patient, rather than on medical evidence (Wolf and Davidovici 2011). In the present study, a 100% cure rate was obtained at all three studied groups after one month of treatment what suggest that all applied modalities were equally efficacious. However, permethrin cream has more rapid onset of action as at the first follow up, the patients from permethrin group reported better improvement than the patients included to the crotamiton and sulfur ointment group. Permethrin is a first-line acaricide in many countries due its high effectiveness against mites and low mammalian toxicity. One treatment with permethrin cream is usually effective in eradicating scabies, but some experts recommend retreatment one week later (Strong and Johnstone 2007). The significant limitation in the use of permethrin in treatment of scabies is its cost, as it is the most expensive drug of all topical scabicides (Roos et al. 2001). Crotamiton (crotonyl-N-ethyl-o-toluidine) as 10% lotion or cream is approved for use in adults with scabies (Pourhasan et al. 2011). The best results seem to be obtained when the drug is applied twice daily for five consecutive days after bathing and changing clothes. Goldust et al. (2014) demonstrated that such application of crotamiton was as effective as single dose of invermectin at two-week follow up. However, some authors do not recommend crotamiton because of the lack of efficacy and toxicity data (Meinking 1999). Sulfur, used as an ointment (2%–10%), is the oldest scabicide in use (Karthikeyan 2005). Topical sulphur ointment is messy, malodourous, stains clothing, and in hot and humid climate, it may lead to irritant dermatitis (Taplin and Meinking 1988). However, it has the advantage of being very cheap and may be the only alternative at patients whose financial state dictates the choice of this antiscabietic drug due to its low cost. Moreover, it is recommended as a safe agent in a treatment of infants, children and pregnant women (Chosidow 2012). Sharquie et al. (2012) evaluated the therapeutic regimen of 8% and 10% topical precipitated sulfur in petrolatum ointment for single day, three successive nights or three successive days at total 97 patients with scabies and they revealed that single-day application was much less effective than three-days treatment. In the group of participants treated for single day, only 42.4% participants responded to management, while in group who received sulfur ointment for three successive nights the response was observed in 90.6% and in those who received the treatment for three successive days it was 96.9% of patients. However, at patients who received only single dose of sulfur ointment fewer side effects were observed. In our study, 10% sulfur ointment was effective at 6 of 18 patients after two-weeks treatment and in remained 11 of patients after three weeks of daily application. Although this drug is well tolerated in most of the patients the major problem of the topical scabicides, life sulfur ointment, is the requirement for repeated application because of their relative low efficacy. On the other hand, the cost is still the lowest.

Our study also evaluated the safety of application of various topical agents in treatment of scabies. Oberoi et al. (2007) investigated the effect of topical application of 1% lindane lotion and 5% permethrin cream on oxidant-antioxidant balance in blood of patients with scabies and they found that permethrin, in contrary to lindane, showed no significant alteration in oxidative stress markers. The medication applied at studied patients of all three groups were well-tolerated by most of the patients and no serious adverse effects were observed in the course of treatment suggesting that all studied agents are safe and non-toxic.

## Conclusions

The topical application of 5% permethrin cream, crotamiton lotion and 10% sulfur ointment was equally efficacious at one-month follow up. Nevertheless, complete clearance of scabietic lesions occured earlier in pertmehrin-treated group than in crotamiton and sulfur groups. No side effects and no re-infections were observed both after administration of permethrin and crotamiton. Although permethrin seems to be the most effective drug in the treatment of scabies, its administration is much more expensive for patients that other common creams. Thus, we conclude that crotamiton may be a cost-effective alternative to permethrin with acceptable cure rate in the treatment of *Sarcoptes scabiei* infection.
